# Submerged vascular anastomosis. A technique for vascular suturing in experimental microsurgery

**DOI:** 10.1590/ACB360807

**Published:** 2021-10-08

**Authors:** Balduino Ferreira de Menezes, Fausto Viterbo de Oliveira, Murilo Sgarbi Secanho, Laísa Brandão Carvalho, Weber Ribolli Moragas, Matheus Scuracchio Fernandes

**Affiliations:** 1 MD, Resident. Department of Plastic Surgery - Hospital das Clínicas - Faculdade de Medicina de Botucatu – Botucatu (SP), Brazil.; 2 PhD, Full Professor. Department of Plastic Surgery - Hospital das Clínicas - Faculdade de Medicina de Botucatu – Botucatu (SP), Brazil.; 3 MD, Resident. Department of Plastic Surgery - Hospital das Clínicas - Faculdade de Medicina de Botucatu – Botucatu (SP), Brazil.; 4 Graduate student. Faculdade de Medicina de Botucatu – Botucatu (SP), Brazil.

**Keywords:** Surgery, Plastic, Microsurgery, Models, Animal, Rats

## Abstract

**Purpose:**

To evaluate the impact of submersion of the microsurgical anastomosis suture area using saline (0.9% NaCl) in an experimental laboratory during the training of medical students and resident physicians.

**Methods:**

Wistar rats (n = 10) were selected to have the two femoral arteries sectioned and anastomosed end-to-end under optical magnification. They were randomly divided, so that on one side suturing was performed under submersion with saline, and the contralateral side was kept dry during the procedure. The surgical times, as well as the patency within 30 min and 72 h of the procedure, were evaluated.

**Results:**

Six male Wistar rats survived the surgical anesthetic procedure, with the average initial weight of 243.3 g and the average artery diameter of 0.86 mm, with average time of 15.67 min for the submerged technique and 20.50 min for the dry technique (p = 0.03). The failure rates were 17 and 50% for the submerged group and the dry one, respectively (p = 0.62).

**Conclusions:**

Submerged microvascular suture does not compromise the patency of the vessel or increase the time of anastomosis. Therefore, it is a strategy that can be applied by the surgeon according to his/her technical preferences.

## Introduction

Microsurgery is highlighted in the scope of plastic surgery. With this technique, the surgeon can transfer tissues to correct complex wounds. This branch of surgery has become increasingly necessary with the evolution of cancer treatments and changes in the profile of trauma mechanisms^1^^,^ 2. This subspecialty is particularly useful in plastic surgery since in school health services it is up to reconstructive surgeons to solve these challenges^2^.

It is based on optical magnification, generally using a surgical microscope, primarily to assist in the anastomosis of small blood vessels^3^.

The technical challenge is high and requires not only great theoretical knowledge, but also high degree of both clinical and experimental practice. When feasible, the latter is important for establishing a solid and secure basis for the surgeon during training^4^^,^ 5.

Over the past 50 years, significant data has been published in specialized journals on ways to create an environment or routine training for surgeons from different specialties including plastic, neurosurgery, orthopedics, and others^6^. From the creation of training models that avoid the use of animals to materials that replace surgical wires, comfort, evolution, and practicality of these procedures are always sought, for better results during training and clinical application in humans^7^^,^ 8.

Methods to achieve adequate vascular patency have been widely researched. It is understood that the success rate should be approximately 100% in an experimental model^9^. Some services use experimental microsurgery as a gradual step before the clinical execution of microsurgical flaps^10^.

Training with nonbiological microsurgery simulators brings immense benefits, including the absence of the need for animal models and ethical implications involved, as well as the flexibility and portability of some equipment^11^. However, the animal model still closely resembles human surgery, making it a step that adds considerable safety to the surgeon and increases the chances of performing efficient anastomoses^12^^,^ 13.

Practice is essential, and most studies show how the quality of anastomoses is directly proportional to the training time^14^.

Anastomosis success rates are expected to be close to 100%. However, these values vary greatly depending on experience and training time^5^.

One of the main steps during training is the preparation of the vessel that receives the anastomosis. Simultaneously, proper manipulation of the adventitial layer of the vessels, particularly arteries, is essential, which must be released to avoid anastomotic failure, as well as being useful for vessel movement during dissection and suturing^15^.

Another important factor when attempting to create an effective anastomosis, that is, with adequate flow and without failure (thrombosis), is to avoid transfixing the layer opposite to the suture. For this, the Carrel technique^16^ was raised, and other strategies have been developed, such as the proper use of forceps and passage of sutures under direct visualization^17^.

It was observed that, during training performed in the laboratory, the adventitious layer of the artery was better exposed, and the vessel was more stable to prevent it from being transfixed when submerged in saline. The following experiment was proposed to assess whether this procedure interferes in patency rate or anastomosis time.

## Methods

This work is according to the rules of Animal Ethics Committee of the Faculdade de Medicina de Botucatu and was approved by the registration number 1,373/2020.

To focus on training and comparison of two microsurgical anastomosis techniques, 10 Wistar rats (all male, aged 60–90 days old) were selected. Their femoral arteries were randomly divided to correspond to two distinct groups. Surgeries were performed in December 2020.

### Experimental laboratory

All activities were performed in the experimental laboratory of the Faculdade de Medicina de Botucatu. In this location, there is the availability of a bioterium, basic microsurgical material (Microsuture^®^), and microscopes (DFV®) with magnification up to 40 times.

Animals were kept in different cages, with water and food *ad libitum*, with air exhaustion, light cycles, and temperature control, in addition to materials for environmental enrichment.

At the end of the procedure, the rats were sacrificed, and the respective carcasses were kept frozen at -20°C for further experimental training. When necessary, the carcasses were incinerated.

### Schedule

The project was divided into two intervention periods. The first one involved the random division of the femoral arteries to separate the groups and perform the initial surgery. In the second, after 72 h, the final patency of the anastomosis was checked.

Initial data included weight, vessel diameter at the section point, surgical time for end-to-end arterial anastomosis, and the number of sutures. The final data included weight and patency assessment after 72 h.

### Groups

The first group corresponded to femoral arteries sutured using a submerged technique, that is, both sides of the sectioned arteries were immersed in 0.9% saline.

The second group corresponded to the femoral arteries that were sutured without the presence of 0.9% saline in the operative field.

### Anesthesia

Combined anesthesia using ketamine and xylazine intraperitoneally was chosen, at doses of 80 and 10 mg/kg, respectively, associated with lidocaine for application at the incision sites at a dose of 7 mg/kg. For intra- and postoperative analgesia, tramadol and dipyrone were subcutaneously administered at doses of 5 and 100 mg/kg, respectively. Dipyrone was reapplied daily for pain control until the second procedure and later completion of the animal with thiopental at a dose of 120 mg/kg.

### Surgery

After initial weighing and trichotomy of the bilateral inguinal region, an oblique incision was made in the inguinal region of the rat, with opening and dissection by planes until the identification of the femoral vessels. The femoral artery was dissected and isolated at its most proximal and largest caliber, with a blue ribbon under it for better contrast. At this point, the adventitial layer was removed, and the diameter of the artery was measured at the point in which it was sectioned. Before the section, a double metallic clamp was positioned proximally and distally to this point. After sectioning, when the group was submerged, the suture region was irrigated with saline, and interrupted suturing was initiated using 10-0 mono nylon, with 3/8 and 0.65 cm cylindrical needles. The same occurred with arteries in the dry group. After the end-to-end suture was timed and the number of sutures was counted, it was waited 30 min to check the patency and then proceeded to approximate the muscle, subcutaneous tissue, and skin of the rat using 5-0 mono nylon sutures.

After 72 h, rats were again subjected to anesthesia. After weight measurement, the vascular suture was reexplored, and patency was checked.

### Patency test

Patency was always verified by an independent examiner. Patency at 30 min and 72 h was verified by the technique proposed by Acland. Two forceps were placed distally to the anastomosis, and a maneuver was made to empty the central region at both sites. Subsequently, the proximal forceps were released to refill the central space. If the created intravascular space were immediately filled with blood in the proximal to distal direction, the patency (flow) was considered successful.

Vessel staining and pulsation were considered indirect signs of successful patency (flow).

The absence of flow after 30 min or 72 h was considered an anastomosis failure.

### Statistical analysis

The data were tabulated using Microsoft Excel^®^. Wilcoxon test was used to assess quantitative variables, while McNemar’s test was used for qualitative variables.

## Results

### Animal and vessel characteristics

Rats had the initial weight varying between 207 and 292 g, with the average of 243.3 g. After 72 h, the average weight increased by 5 g (Table 1). Both right and left femoral arteries varied from 0.8 to 0.9 mm in diameter, with equal averages of 0.86 mm.

**Table 1:** Comparison between initial and final weights.

Rat	Initial Weight (mg)	Final Weight (mg)
1	236	232
2	232	219
3	292	300
4	254	262
5	207	229
6	239	248

Regarding the number of sutures by anastomosis, the first group had the average of 5.5 on the right and 4.83 on the left, with a variation on the right and left from 5 to 7 and 4 to 6, respectively.

### Surgical time and anastomosis patency or flow

Regarding the time to perform anastomosis, in the first group, the average was 15.6 min when the submerged suture technique was performed, against 20.5 min using the dry technique (p = 0.03). Points per minute were compared, and it was better in the submerged group than in the other groups (p = 0.16), as shown in Table 2.

**Table 2:** Mean and standard deviation for variables according to group with statistical analysis.

Variables		Group		p
		Submerged	Dry	
Diameter	Mean	0.87	0.87	1.00
	SD	0.05	0.05	
Surgical time	Mean	15.67	20.50	0.11
	SD	5.28	4.09	
Stitches/ minute	Mean	0.34	0.26	0.07
	SD	0.10	0.02	

SD: standard deviation.

Patency was successful in all the rats within the first 30 min. After 72 h, final patency was 83% of the anastomoses using the submerged technique, and 50% when performed using the dry technique (p = 0.62), as seen in Table 3.

**Table 3:** Failure rate in anastomosis by group with statistical analysis.

Submerged	Dry	p
0.17	0.50	0.22

### Complications

Rat number 2 in the first group had a local hematoma. Four rats did not survive the anesthetic procedure and were excluded from the analyses, because there were no other intraoperative complications that justified the death (Table 4).

**Table 4:** Summary of results related to vessel diameter, stitches by anastomosis, surgical time, group and final patency/flow.

Rat	Diameter - Right artery (mm)	Diameter - Left artery (mm)	Stitches - Right artery	Stitches - Left artery	Time – Right artery (min)	Time – Left artery (min)	Group – right artery	Group – right artery	Final flow – right artery	Final flow – left artery
1	0.8	0.8	5	5	16	18	Submerged	Dry	Successful	Successful
2	0.9	0.9	7	5	28	26	Dry	Submerged	Fail	Fail
3	0.9	0.9	5	5	14	20	Submerges	Dry	Successful	Fail
4	0.9	0.9	6	6	22	12	Dry	Submerged	Successful	Successful
5	0.8	0.8	5	4	14	17	Submerged	Dry	Successful	Fail
6	0.9	0.9	5	4	18	12	Dry	Submerged	Successful	Successful

**Figure 1:**
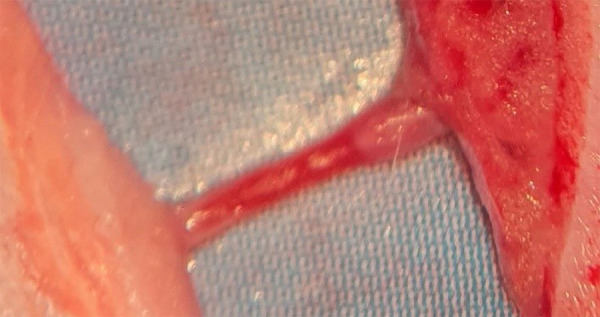
The dry vessel at the beginning of the dissection.

**Figure 2:**
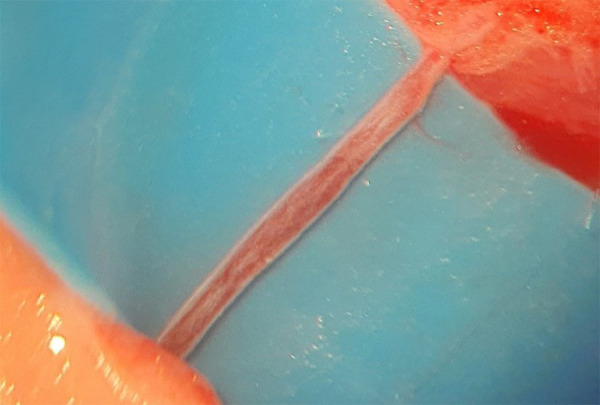
The submerged vessel at the beginning of the dissection, in which the adventitia layer is more easily identified.

**Figure 3:**
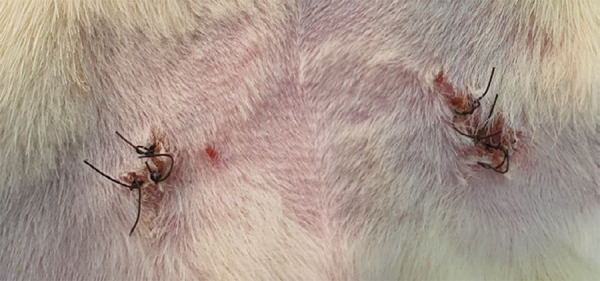
Appearance of the surgical site immediately before the second intervention to assess the final patency.

**Figure 4:**
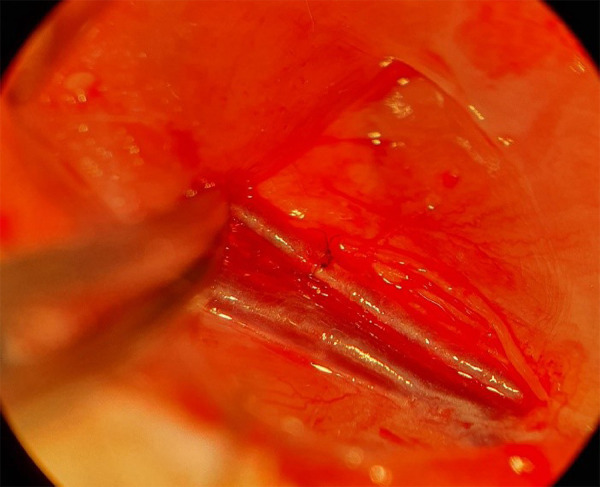
Femoral artery is dissected and prepared for the final patency test using the Acland technique (refill test).

Figure 1 shows the dry vessel, compared to the submerged vessel in Fig. 2, in which the adventitia layer is more easily identified. Figure 3 reveals the appearance of the surgical site immediately before the second intervention to assess the final patency. Figure 4 demonstrates that the femoral artery is dissected and prepared for the final patency test using the Acland technique (refill test).

## Discussion

Microsurgical anastomosis in small-caliber vessels requires intense and focused training, as assessed in several previous studies. The concept of microsurgery arises when vessels smaller than 2 mm are approached, and with current advances there is already a subdivision, super microsurgery. In this case, vessels have gauges smaller than 0.5 mm and greatly expand their clinical applicabilities^18^.

An initial doubt regarding the submerged technique was the possible increase in the surgical time since the liquid could impact the refraction of light and even impair the visualization of the vessel, as well as the memory and malleability of the mono nylon thread by the presence of saline. In practice, it did not happen, since the surgical time with this technique was 30% lower with statistical significance (p = 0.03). This occurred because, despite the aforementioned interferences, saline provides a sensation of stability of the posterior wall and prevents it from being reached during the passage of sutures, causing one of the most common technical errors, transfixation of the posterior wall of the blood vessel.

Several studies have shown the importance of achieving high patency rates, generally approximately 95% of success, during experimental training before clinical treatment in humans. However, although the found patency rates are lower than this number, they are considered satisfactory because the average diameter of the vessels covered in this study was smaller than the one of many experiments with success rates close to 100%^19^.

The average time spent in each anastomosis is not well established in the literature, as it varies according to the number of sutures and vessel caliber, among other factors. The average time of anastomosis in the present study was considered satisfactory as it was relatively shorter when compared to other studies, despite a smaller number of points in each anastomosis^20^.

The decision for interrupted or continuous sutures during anastomosis is still dependent on the surgeon’s experience and preference. While continuous suture has the benefit of a theoretical time gain and less leakage, it can also cause stenosis and leave more surgical thread in contact with the bloodstream, causing thrombosis. In contrast, interrupted sutures may be more useful when there is a difference between the caliber of the vessels^21^.

While some studies evaluated patencies only in the first surgical period, generally immediately, at 5 and 30 min, others, like the present one, reevaluate them later, at intervals ranging from days to weeks^6^^,^ 9. An early reassessment in 72 h was preferred, and not in one week, due to the risk of increasing animal suffering, with little statistical benefit for the final result of the experiment^22^.

Despite the emergence of new materials and technologies that attempt to simulate training in experimental microsurgery, the model with live animals remains the gold standard. Although these materials are close to the real ones, or the cadaverous models maintain the same physical structure, the simulation is not comparable with the live model^23^. Due to the availability of the use of this model in our institution, it is believed that, although it provides a small sample for this study, it would still provide the most realistic results.

As the surgeons were still in the initial training phase, only arterial sutures were performed, while the venous approach was avoided.

Establishing surgical tactics and techniques that enable the procedure to be performed more easily can stimulate the continuity of training and improve the general quality of the microsurgeon during training.

## Conclusion

The experimental microsurgical suture technique performed in a submerged form does not compromise its patency nor increases the anastomosis time, and it is a tactic that can be applied by the surgeon according to his/her technical preferences.
